# Ridge preservation using an in situ hardening biphasic calcium phosphate (β-TCP/HA) bone graft substitute—a clinical, radiological, and histological study

**DOI:** 10.1186/s40729-017-0086-2

**Published:** 2017-06-22

**Authors:** Ashish Kakar, Bappanadu H. Sripathi Rao, Shashikanth Hegde, Nikhil Deshpande, Annette Lindner, Heiner Nagursky, Aditya Patney, Harsh Mahajan

**Affiliations:** 1 Yenepoya University Dental College, University Road, Mangalore, 575018 India; 2 Dental Foundations and Research Centre, Malad, Mumbai, 400064 India; 3 Department of Oral and Maxillofacial Surgery, Center for Dental Medicine, Medical Center—University of Freiburg, Hugstetter Str. 55, 79106 Freiburg, Germany; 4 Mahajan Imaging Center, K-18 Hauz Khas Enclave, New Delhi, 110016 India

## Abstract

**Background:**

Post-Extraction ridge preservation using bone graft substitutes is a conservative technique to maintain the width of the alveolar ridge. The objective of the present study was to evaluate an in situ hardening biphasic (HA/β-TCP) bone graft substitutes for ridge preservation without primary wound closure or a dental membrane.

**Methods:**

A total of 15 patients reported for tooth extraction were enrolled in this study. Implants were placed in average 5.2 ± 2 months after socket grafting. At this visit, Cone Beam CT (CBCT) images and core biopsies were taken. Implant stability (ISQ) was assessed at the insertion as well as at the day of final restoration.

**Results:**

CBCT data revealed 0.79 ± 0.73 mm ridge width reduction from grafting to implant placement. Histomorphometric analysis of core biopsy samples revealed in average 21.34 ± 9.14% of new bone in the grafted sites. Primary implant stability was high (ISQ levels 70.3 ± 9.6) and further increased until final restoration.

**Conclusions:**

The results of this study show that grafting of intact post-extraction sockets using a biphasic in situ hardening bone graft substitute results in an effective preservation of the ridge contour and sufficient new bone formation in the grafted sites, which is imperative for successful implant placement.

## Background

Following tooth extraction, the alveolar ridge will decrease in volume and change its morphology [1, 2]. These changes are clinically significant [3] and can complicate the placement of a conventional bridge or an implant-supported crown. Post-extraction maintenance of the alveolar ridge following the principles of ridge preservation using bone graft substitutes minimizes ridge resorption and, thus, facilitate subsequent placement of an implant that satisfies esthetic and functional criteria, limiting the need of additional bone augmentation procedures [4–8].

Bone graft substitutes have been in use for several decades to replace autogenous bone grafts. The first experimental use of calcium phosphates as bone graft substitutes was reported in the 1920s [9]. These materials were recognized early on as promising candidates due to their chemical similarity to native bone, which is mainly composed of calcium phosphate hydroxyapatite (70% of dry weight). Synthetic calcium phosphate ceramics belong to the group of alloplastic biomaterials and are nowadays widely used. Synthetic materials are osteoconductive and free of any risk of transmitting infections or diseases in contrast to animal-derived xenografts or materials from human cadaver (allografts). Synthetic calcium phosphates comprise of resorbable ß-tricalcium phosphates, nonresorbable hydroxyapatites, and combinations of these two materials that are named biphasic calcium phosphate materials (BCP). ß-TCP materials resorb completely and are replaced by the body’s own tissue whereas hydroxyapatite-containing materials do not resorb but are integrated in the host’s bone [10–13].

Both ß-TCP and BCP materials have been successfully used in clinical practice, and their efficacy and safety have been demonstrated in a multitude of clinical studies [14–17]. However, it is still a matter of discussion whether resorbable ß-TCP materials can provide sufficient support to adequately maintain the ridge in contrast to nonresorbable hydroxyapatite-containing BCP materials.

The aim of this clinical, radiological, and histological study was to evaluate the potential of the use of poly(lactide-co-glycolide) acid (PLGA)-coated alloplastic BCP particles to preserve the ridge profile while supporting the formation of new bone in intact socket defect after tooth extraction. The PLGA coating of the BCP granules provide additional initial in situ hardening properties and*,* therefore, allow stabilization of the bone graft substitute in the empty extraction socket without the use of a barrier membrane to stabilize the graft and to cover the defect without achieving primary wound closure [18].

To our knowledge, this is the first systematic clinical, radiographic, and histological evaluation that assesses bone formation and ridge width preservation after socket grafting using an in situ hardening biphasic bone graft substitute in healthy patients.

## Methods

This study was approved by the Yenepoya University Ethics committee, Mangalore, India (Approval Number YOEC83/8/3/2014). Fifteen patients who required extraction of a maxillary or mandibular tooth and subsequent single-tooth implant placement and who met the inclusion and exclusion criteria were included in this prospective single-arm clinical study. The patients (4 females and 11 males) had a mean age of 51.3± 14.8 years (range: 27 to 75 years). The site-specific areas and teeth numbers for the study are shown in Table 1.

All patients were systemically healthy at the time of consultation and study inclusion. The reasons for extraction included endodontic treatment failures and advanced caries lesions and tooth fractures. If more than one tooth was extracted (maximum of three teeth), all teeth were treated but only the most anterior tooth with intact socket wall was selected for the study. Standard exclusion criteria for bone grafting procedures were applied including allergy, systemic chronic disease, alcoholism and drug abuse, pregnancy, or nursing mothers. Smokers and patients with any oral tobacco use/habits were excluded. Patients using dentures were also excluded. Patients with acute abscesses or active infections localized in the proximity of the prospective surgical field and those with heavily scarred mucosa at the site and patients who had malignant diseases or other diseases treated with radiotherapy or chemotherapeutic agents (chemotherapy) during the past 5 years were excluded*.*

### Surgical technique

**Fig. 1:**
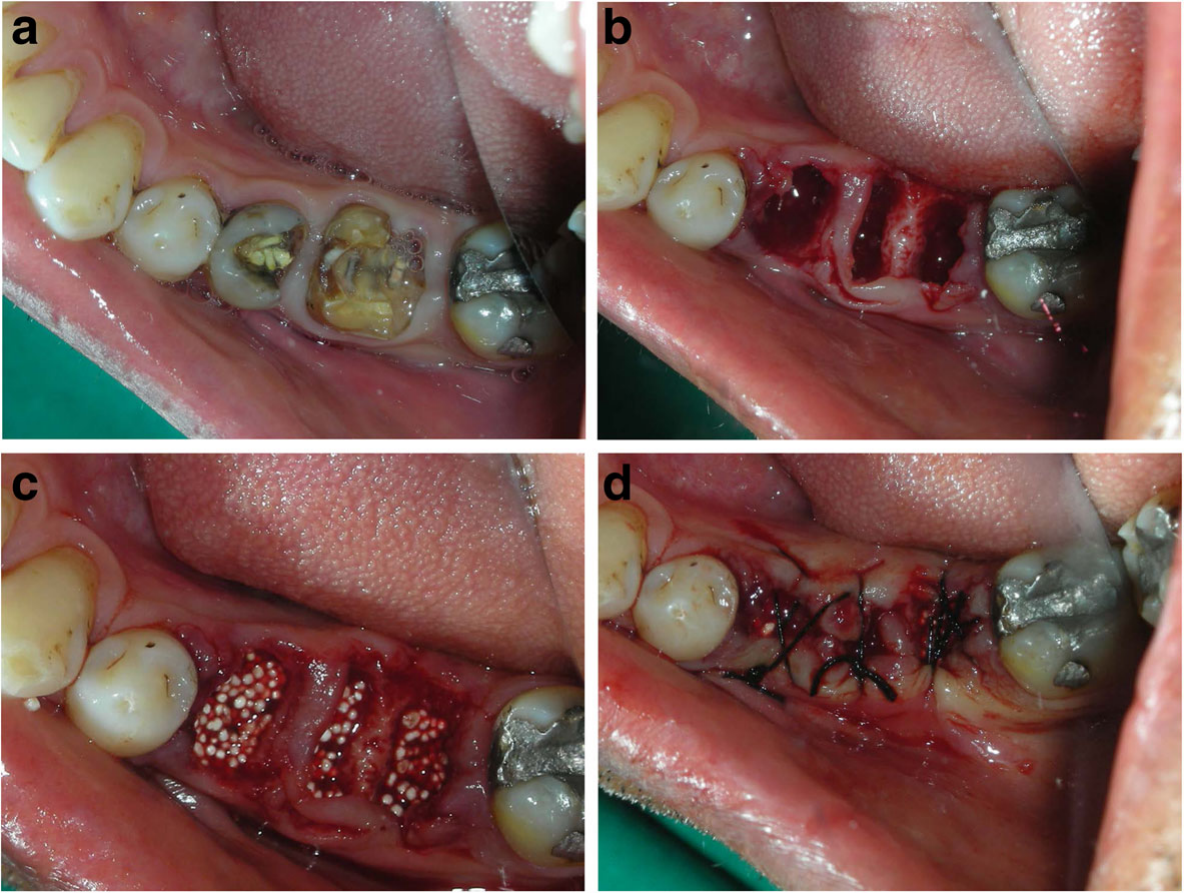
**a** Clinical occlusal view with fractured 45 and 46. **b** Post-extraction view of the socket. Note minimal trauma to the soft tissue and no flap reflection on the surgical site. **c** Graft material condensed into the extraction sockets showing good initial graft stability. **d** Black silk sutures placed with tissue approximation and no releasing incision in the flaps

All surgical procedures were performed by one surgeon in this study. The following procedures were planned for all sites. Tooth extraction was performed under local anesthesia without flap elevation (Fig. 1a, b). Periotomes and luxators (Directa, Sweden) were gently used for all extraction procedures. Extraction forceps were only used when the tooth was mobile in the extraction socket. All multi-rooted teeth were sectioned with a Lindemann burr (Komet Inc., Lemgo, Germany) under copious irrigation with sterile saline to minimize extraction trauma. Each root of the multi-rooted teeth was independently mobilized and carefully luxated. Attention was given not to damage the surrounding soft and hard tissues, especially in the buccal aspect. All sockets were thoroughly curetted to remove granulation tissue, followed by irrigation and rinsing with sterile saline. A ball-ended probe was then utilized to explore the buccal plate. All teeth included for this study had an intact buccal and lingual plate (four-wall post-extraction sockets). A biphasic alloplastic in situ hardening bone graft substitute (GUIDOR easy-graft CRYSTAL, Sunstar Suisse SA, Etoy, Switzerland) was used to graft the site according to the manufacturer’s instructions. Attention was given not to overfill the extraction socket to avoid any displacement of the entire graft mass after mechanical irritation during the first phases of healing. A saline-wet gauze was used to further compact the granules and accelerate the hardening of the graft in situ so that after a few minutes the alloplastic bone substitute formed a stable, solid, porous scaffold for the host osseous regeneration (Fig. 1c). An interrupted tension-free nonresorbable 4-0 sutures (Black Silk, Ethicon, Johnson & Johnson, Somerville, NJ, USA) was placed over the filled socket to achieve soft tissue stability (Fig. 1d). All sites were left uncovered without obtaining primary closure in order to heal by secondary intention. The patients did not wear any prosthesis during the healing period.

**Fig. 2:**
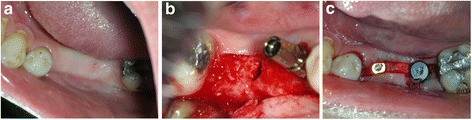
**a** Clinical postoperative view after 4 months. Note that the healing was achieved only with tissue approximation. A good width of keratinized tissue is visible along with ridge preservation. Ready for implant placement in the grafted areas. **b** Implant placed in 45 area. Core biopsy sample taken from area 46. Note the integration of graft particles in the preserved alveolar ridge also inside the osteotomy site of 46. **c** Two Xive (Dentsply) implants placed in the preserved ridge. **d**. Postoperative X ray showing the implant positions in the mandible where the teeth were extracted and ridge preservation was accomplished

**Fig. 3:**
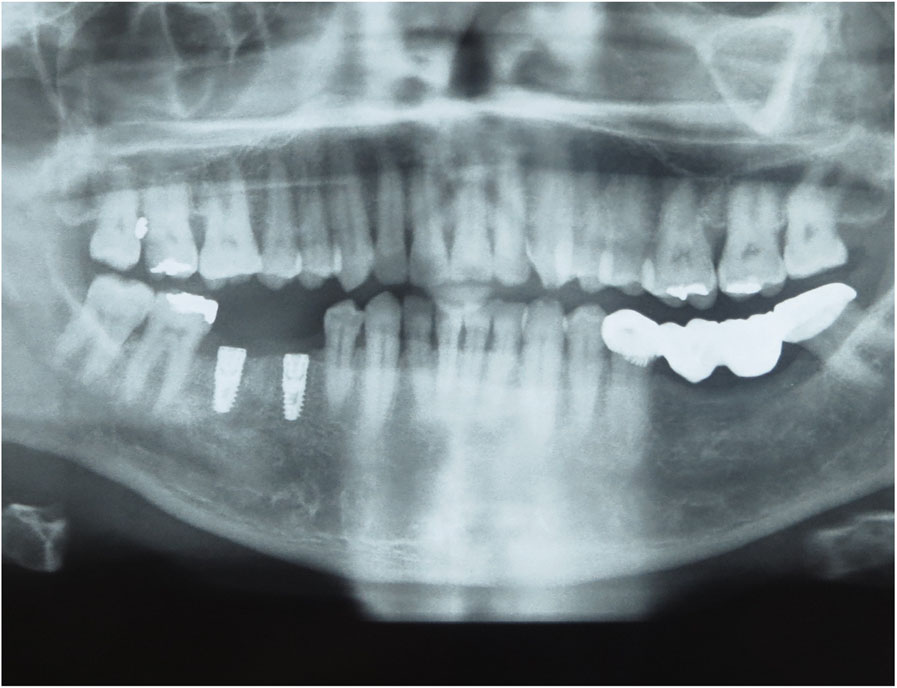
Postoperative X ray showing the implant positions in the mandible where the teeth were extracted and ridge preservation was accomplished

**Fig. 4:**
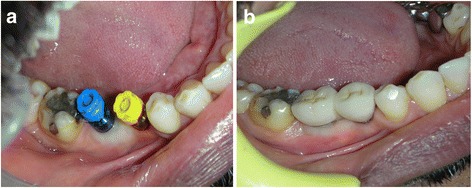
**a** Second stage surgery followed by impression making. Note the excellent width of keratinized tissue which was also preserved. **b** Implant crowns placed and loaded after 3 months of placement

Antibiotic therapy consisting of 1 g amoxicillin every 12 h for 4 days and mouth rinsing with 0.2% chlorhexidine every 8 h for 10 days were prescribed. The suture was removed 1 week postoperatively. After 3 to 8 months (average 5.2 ± 2 months), the sites (Fig. 2a) were reentered for implant placement. A site-specific full thickness mucoperiosteal flap was elevated to expose the regenerated hard tissue. A bone core biopsy was taken with a minimum depth of 7 mm from the center of the site using a trephine drill with a diameter of 2.3 mm (Komet Inc., Lemgo, Germany) (Fig. 2b). Following the harvesting of the bone sample, the preparation of the bony bed was completed at the same site and a dental implant was placed (Fig. 2b, c) according to the manufacturer’s surgical protocol. Immediately after placement, the initial stability was measured by resonance frequency analysis (Osstell ISQ, Gothenburg, Sweden). For each implant, two ISQ measurements were recorded, palatally (or lingually) and bucally, according to the guidelines of the company. The measurement was repeated after placement of the final restoration 4 months after implant placement. Average of the ISQ measurements was then reported. The mucoperiosteal flap was closed with interrupted nonresorbable 4-0 sutures (Silk, Ethicon, Johnson & Johnson, Somerville, NJ, USA), and Fig. 3 shows the postoperative radiograph of the implants placed in the preserved ridge. Figure 4a shows the second surgery followed by impression making, and Fig. 4b shows implant crowns placed and loaded after 3 months of placement.

### Assessment of ridge width changes by cone beam computer tomography

**Fig. 5:**
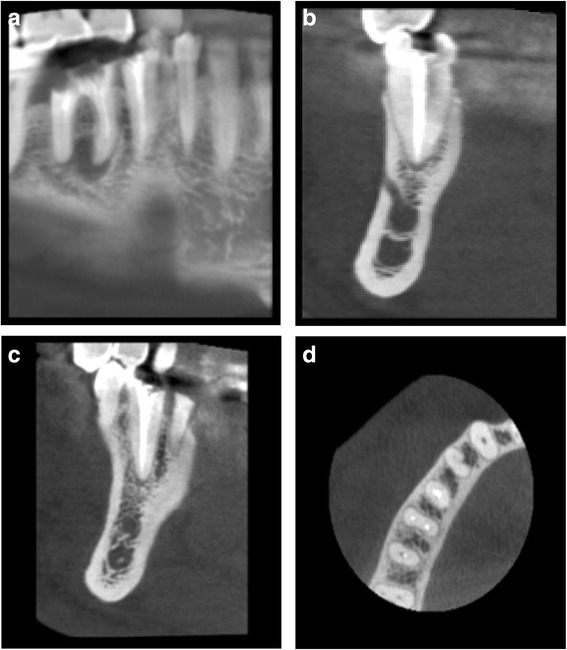
CBCT images of the extraction site. **a** Preoperative CBCT showing fractured and un-restorable teeth #45 and #46 planned to be extracted. **b**–**d** Cross sectional views

**Fig. 6:**
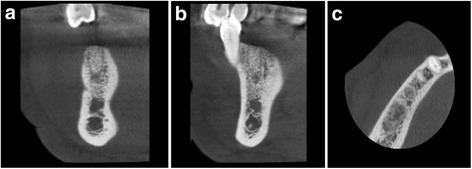
**a**–**c** Four-month postoperative CBCT showing graft integration and preservation of ridge without collapse of the buccal or lingual cortical plates also showing the cross sections in the grafted area

The area of interest (site of socket preservation and grafting) was identified in accordance with the site which was grafted (Fig. 5a–d). Axial correction of the view was performed in conformity with angulation of the alveolar ridge. Contours of the crestal bone were identified, considering apical recession of the alveolar crestal level, if any on buccal or lingual/palatal aspect. Buccolingual/palatal width of the alveolus was then determined 2 mm below the alveolar crestal level before tooth extraction and at the time of implant placement. Ridge width changes were calculated by subtracting preoperative measurements from postoperative ridge width measurements (Fig. 6a–c).

### Histological and histomorphometric evaluation

**Fig. 7:**
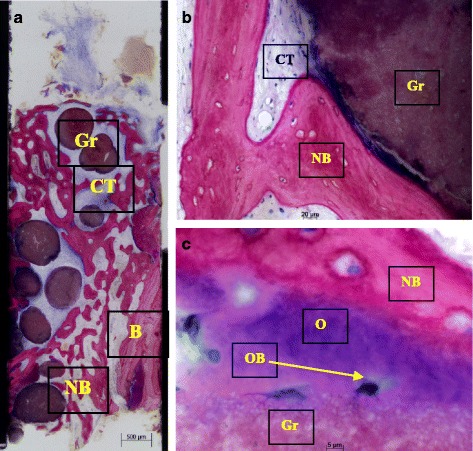
**a**–**c** Histological sections of bone core biopsy taken from the site of implantation using a trephine bur. **a** Overview image of coronal-apical cut through the entire core biopsy showing formation of new bone (*NB*) next to old bone of the extraction socket (*B*). easy-graft CRYSTAL particles (*Gr*) are embedded in well perfused connective tissue (*CT*) and new bone (*NB*) (Azur II and Pararosanilin, original magnification ×50). **b** Integration of easy-graft CRYSTAL particle (*Gr*) into newly formed bone (*NB*) and connective tissue (*CT*) showing tight contact between graft particle and new bone. **c** High magnification (×200) images of the interface between graft particle (*Gr*) and new bone (*NB*) showing osteoblasts (*OB*) forming osteoid (O) and formation of new bone (*NB*) on the surface of easy-graft CRYSTAL particles (*Gr*) (Azur II and Pararosanilin, original magnification ×200)

Bone biopsies were harvested using a trephine bur at the site of implant placement. The trephine burs including the bone biopsies were fixed in 4% formalin for 5–7 days, rinsed in water, and dehydrated in serial steps of ethanol (70, 80, 90, and 100%), remaining for 1 day in each concentration. Specimens were then infiltrated, embedded, and polymerized in resin (Technovit 9100, Heraeus Kulzer, Wehrheim, Germany) according to the manufacturer’s instructions. After polymerization, samples were cut in 500-μm sections using a precision cutting machine Secotom 50 (Struers, Ballerup, Denmark). The sections were mounted on acrylic slides (Maertin, Freiburg, Germany) and ground to a final thickness of approximately 60 μm on a rotating grinding plate (Struers, Ballerup, Denmark). Specimens were subsequently stained with Azur II and Pararosanilin (Merck, Darmstadt, Germany), which allowed for a differentiation between graft granules, and preexisting and newly formed bone. Imaging was performed with an Axio ImagerM1 microscope equipped with a digital AxioCamHRc (Carl Zeiss,Göttingen, Germany). Histomorphometric analysis was performed by one observer digitally using the analySIS FIVE software (Soft Imaging System, Munster, Germany). The reference area for the histomorphometric evaluation was the entire area in the biopsy excluding old bone from the defect margins. Values measured in percent of the examined area were taken for remnants of graft material, newly formed bone, and soft tissue/bone marrow (Fig. 7a–c).

### Statistics

Data were expressed as means ± standard deviations (SD). A two-sided paired *t* test was used to assess significance of intra-group longitudinal changes for ridge width changes and changes of ISQ. Statistical significance was set to *p* < 0.05 for a two-sided test.

## Results

Fifteen patients (4 females and 11 males) with a mean age of 51.3 + 14.8 years (range: 27 to 75 years) participated in this randomized clinical trial. The site specific areas and teeth numbers for the study are shown in Table 1.

**Table 1:** Buccal and palatal ISQ values at implant placement and time of loading

		ISQ level at implant placement			ISQ level at loading		
Patient no.	Tooth no.	Buccal	Palatal	Average	Buccal	Palatal	Average
1	17	67	71	69	70	70	70
2	17	73	72	72.5	76	76	76
3	36	76	78	77	84	80	82
4	26	48	48	48	61	66	63.5
5	25	49	49	49	71	66	68.5
6	37	71	78	74.5	69	78	73.5
7	45	76	74	75	72	74	73
8	36	80	82	81	85	85	85
9	37	74	74	74	72	72	72
10	46	76	72	74	74	74	74
11	27	71	71	71	74	72	73
12	47	72	72	72	74	71	72.5
13	36	71	71	71	76	72	74
14	36	80	82	81	82	82	82
15	17	70	62	66	66	66	66
Mean		70.3	70.4	70.3	73.7	73.6	73.7
Standard deviation		9.5	10.2	9.7	6.5	5.8	5.9

In all cases, the postoperative healing was uneventful. Clinically, the soft tissue healing pattern observed was very similar in all cases. The soft tissue on all sites was completely closed and healed at the 4-month recall period (Fig. 2a). There was no loss or sequestration of bone graft granules observed during the healing period although the sites were left uncovered to heal for secondary intention. Due to the specific in situ hardening biomechanical properties of the grafting material, the stable granules provided a solid immobile scaffold over which newly formed soft tissue migrated from the margins of the sockets, achieving secondary intention soft tissue healing. In the first stage, graft particles were embedded in fibrin matrix that was slowly replaced by a layer of connective tissue that slowly covered the occlusal aspect of the exposed graft starting from the wound edges. Subsequently, epithelial tissue proliferated from the periphery over the connective tissue layer and after 4 months all areas were completely covered with newly formed keratinized epithelium. At this time point, clinical examination showed that the volume and architecture of the ridges were adequately preserved. Reentry for implant placement was between 3.3 and 8.4 months and on average 5.2 ± 2 months after socket grafting. At this time point, the grafted areas were filled with newly formed bone with particles of the bone graft substitute (BGS) visible in the regenerated hard tissue. All implants were placed at the precise 3D position showing excellent initial stability, and the potential implant sites had a good resistance to drilling clinically. At implant placement, the mean final ISQ measurements were 70.3 ± 9.6 (Table 1) showing excellent initial stability for the majority of the cases.

Cone beam computer tomography (CBCT) was performed before tooth extraction and at the time point of implant placement. Mean ridge width reduction before tooth extraction to implant placement was calculated to effect in 0.79 ± 0.73 mm horizontal bone loss (Table 2). Primary implant stability was achieved in all 15 cases, showed in average high ISQ levels 70.3 ± 9.7 (buccal/palatal), and further increased until final restoration to an average level of 73.7 ± 6.0 (Table 3) showing commenced osseointegration of the dental implant in the grafted sites.

**Table 2:** Width ridge changes assess by cone beam computer tomography (CBCT)

Patient no.	Tooth no.	Ridge width baseline [mm]	Ridge width implant placement [mm]	Ridge width changes [mm]
1	17	8.2	7.4	0.8
2	17	12.6	12.3	0.3
3	36	12	11.6	0.4
4	26	8.4	8.2	0.2
5	25	7.5	7.2	0.3
6	37	9.4	6.2	3.2
7	45	8.4	7.9	0.5
8	36	13.3	12.8	0.5
9	37	14.2	13.6	0.6
10	46	16.2	14.8	1.4
11	27	14.2	13.6	0.6
12	47	12.6	11.8	0.8
13	36	13.6	12.7	0.9
14	36	17.4	16.9	0.5
15	17	9.6	8.7	0.9
Mean		11.84	11.05	0.79
Standard deviation		3.10	3.22	0.73

**Table 3:** Histomorphometric evaluation of core biopsy sections

Patient no.	Gender	Patient’s age	Tooth no.	Time post extraction [month]	% New bone	% Residual graft	% Connective tissue
1	M	48	17	8.4	17.9	31.3	50.8
2	F	68	17	8.4	23.5	17	59.4
3	M	54	36	3.9	17	25.8	57.2
4	M	39	26	4.5	22.7	26.3	51
5	F	40	25	4.5	14.3	30.7	55
6	M	50	37	4.5	21.1	26.6	52.2
7	F	32	45	4.0	39.4	16	44.6
8	M	55	36	3.8	9.6	20.2	70.2
9	M	44	37	3.3	12.8	22.9	64.3
10	F	27	46	5.1	22.5	25.7	51.8
11	M	76	27	5.7	9.6	41.6	48.8
12	M	74	47	4.6	34.3	23.8	41.9
13	M	75	36	8.0	25.6	28.6	45.8
14	M	74	36	4.4	35	9.6	47.6
15	M	47	17	5.0	14.8	46.8	54.4
Mean				5.2	21.3	26.2	53.0
Standard deviation				2.0	9.2	9.4	7.5

Histologically, all analyzed biopsies contained newly formed bone, residual graft material, and well vascularized uninflamed connective tissue (Fig. 7a–c). No necrosis or foreign body reactions were detected. The graft granules were in contact with active osteoblasts forming osteoid and new woven bone, demonstrating persistent osteogenesis. Active cellular resorption of the BCP particles was not observed. In many areas, active bone remodeling was evident with mature lamellar bone replacing the woven bone. Histomorphometric analysis revealed that the grafted site was occupied with an average of 21.34% ± 9.14% new bone, 26.19 ± 9.38% residual graft material, and 53% ± 7.51% connective tissue (Table 3). In all cases, 3–4 months after implant placement, a vertical crestal incision was made and a healing abutment was placed. After allowing 2 weeks for the maturation of the soft tissues, the final restoration was fabricated with successful functional and esthetic results.

## Discussion

Ridge preservation following dental extractions is fundamental, preserving the ridge profile for subsequent implant placement and providing a sustained function and esthetics. This clinical trial reports on the successful application of an in situ hardening biphasic alloplastic bone graft substitute for ridge preservation and subsequent implant placement in 15 healthy patients. A routine but minimally invasive surgical protocol was followed in all cases. Extractions and grafting were performed without raising a flap. After grafting, the augmented sites were not covered with a barrier membrane or an advanced buccal flap for primary closure. Elevating the periosteum from the buccal bone to create a mucoperiosteal flap can compromise the blood supply of the exposed bone surface, leading to osteoclastic activity and increased bone resorption [19]. Moreover, this approach was selected in order to minimize patient morbidity, surgical time, and cost, but mostly in an attempt not to displace the mucogingival junction and to allow for the spontaneous formation of new keratinized soft tissue over the grafted site. According to the clinical findings of this report, it seems that secondary intention soft tissue healing of grafted post-extraction sites can be achieved when filling the sockets with in situ hardening bone graft substitutes without the use of barrier membranes. The findings and parameters for soft tissue healing are in alignment with observations previously reported on in situ hardening β-TCP materials [18] as well as in situ hardening biophasic calcium phosphate (BCP) materials [20]. Both authors have reported excellent and uneventful soft tissue healing without achieving primary closure or covering the site with a barrier membrane or soft tissue punch. The coating of calcium phosphate granules with PLGA results in a stable, solid alloplastic bone substitute, deterring the loss of exposed granules. In contrast, a flap and/or a barrier membrane may be necessary to provide the necessary stability and protection for loose particulate bone grafts without self-hardening characteristics. Furthermore, the approach using reflection of a mucosal flap brings significant disadvantages as elevation of the periosteum from the bone will compromise the vascular supply to the bone surface leading to osteoclastic activity and increased bone resorption [21, 22]. After analyzing the existing evidence, the authors concluded that achieving primary closure did not present beneficial effects on preserving the ridge width. On the other hand, patients experience more discomfort and the mucogingival junction was significantly more coronally displaced when primary flap closure was achieved compared to secondary intention healing [23–25].

As previously reported, secondary intention soft tissue healing of grafted post-extraction sites can be well achieved when using an in situ hardening and in situ stabilizing bone graft substitutes without the need of a dental membrane [18, 20]. Findings of the present report corroborate these results. The authors found that all sites healed uneventfully with coverage of soft tissue and no local complications. In the present study, all sites were fully covered with newly formed keratinized soft tissue while the buccal keratinized soft tissues were preserved. As the presence of an adequate zone of keratinized gingiva is an important parameter in achieving esthetic implant restorations [26], preventing future mucosal recessions, and improving the overall long-term implant stability, the use of flapless techniques by taking advantage of the biomechanical properties of in situ hardening alloplastic grafts seems to be a benefit for the clinicians and patients when applicable.

Following tooth extraction, the width of the alveolar ridge seems to undergo pronounced shrinkage which can render subsequent implant placement challenging [5]. In all patients, the dimensions of the ridge were adequately preserved at the time of implant placement (5.1 ± 2 months) and bone resorption was marginal. Mean ridge width reduction assessed by CBCT was 0.79 ± 0.73 mm (Table 2), which is well below the results reported for ridge preservation techniques using both bone substitute material in combination with membranes or soft tissue grafts [26, 27]. A recent meta-analysis [27] further suggested that the choice of the biomaterial did not have a significant influence on the ridge preservation after tooth extraction and that all materials sufficiently maintained the ridge dimensions.

At reentry, all sites were filled with sufficient bone, allowing for the precise placement of an implant at the optimal position. The histomorphometric analysis of the harvested samples revealed pronounced new bone formation. In order to investigate the remineralization of the grafted sites, it is of outmost importance to discriminate between the old and new bone and only assess the new bone that is found within the graft site of the biopsy. Displaying only the total amount of mineralized bone found in the biopsy, including old bone from the defect margins, will falsify the true de novo bone formation and defect fill. In this study, 21.34 ± 9.14% of the grafted area was occupied by newly formed vital bone (Table 1). Similar levels of new bone formation have been reported in rabbit calvaria defects model published by Schmidlin et al. [28] using the same material and revealing 20.16 ± 5.27% of new bone in the defect site after 4 weeks of healing. Moreover, the amount of new bone formation in the grafted site is comparable to that reported for other BCP materials and xenografts used in sinus augmentation [14, 29]. In this histomorphometric study including core biopsies from 38 sinus augmentations, a mean percentage of new bone of 21.6 ± 10.0% was reported for particulate BCP and 19.8 ± 7.9% for particulate xenograft bone substitutes.

Likewise, the reported amount of residual grafting material in the defect site was similar. In average, only 26.2 ± 9.4% of the defect was occupied with residual graft material in this study which is well in line with 26.6 ± 5.2% reported for BCP but below the 37.7 ± 8.5% reported for xenograft [14].

All 15 implants could be placed without the need for additional bone augmentation. ISQ values measured after implant placement revealed adequate primary stability of the implants. It is important to note that primary stability is not only affected by the geometry of the implant placed (i.e., length, diameter, and type) and the placement technique used (relation between drill size and implant size, tapping) but also positively related to the quality, density, and quantity of the local bone [29–31]. Considering that in the present study the same implant type and technique were utilized, the reported high primary stability values might suggest good quality of the regenerated bone at 4 months postoperatively.

## Conclusions

The results of this clinical study support the use of a biphasic in situ hardening alloplastic bone graft substitute for ridge preservation in intact post-extraction sites without the use of a dental membrane. Therefore, grafting of sockets without primary wound closure or using dental membranes or a soft tissue punch can be an effective minimally invasive method of preserving the contour and architecture of the alveolar ridge while supporting sufficient bone formation for implant placement. The hardening characteristics of the grafting material used seem to be of great importance for the stability of the healing site, secondary wound healing, and the success of the above technique. Additional prospective studies using control groups, larger patient populations, and other time frames are needed in order to confirm and supplement the present findings.
